# Unravelling Low-Value Care Decision-Making: Residents’ Perspectives on the Influence of Contextual Factors

**DOI:** 10.34172/ijhpm.2024.7907

**Published:** 2024-04-06

**Authors:** Lotte A. Bock, Cindy Y.G. Noben, Roel H.L. Haeren, Florine A. Hiemstra, Walther N.K.A. van Mook, Brigitte A.B. Essers

**Affiliations:** ^1^Academy of Postgraduate Medical Education, Maastricht University Medical Centre, Maastricht, The Netherlands.; ^2^School of Health Professions Education, Maastricht University, Maastricht, The Netherlands.; ^3^Department of Neurosurgery, Maastricht University Medical Centre, Maastricht, The Netherlands.; ^4^Faculty of Health, Medicine, and Life Sciences, Maastricht University, Maastricht, The Netherlands.; ^5^Department of Intensive Care Medicine, Maastricht University Medical Centre, Maastricht, The Netherlands.; ^6^Department of Clinical Epidemiology and Medical Technology Assessment, Maastricht University Medical Centre, Maastricht, The Netherlands.

**Keywords:** Low-Value Care, Context, Contextual Factors, Decision-Making, Residents, Overuse

## Abstract

**Background:** Several initiatives have been developed to target low-value care (ie, waste) in decision-making with varying success. As such, decision-making is a complex process and context’s influence on decisions concerning low-value care is limitedly explored. Hence, a more detailed understanding of residents’ decision-making is needed to reduce future low-value care. This study explores which contextual factors residents experience to influence their decision-making concerning low-value care.

**Methods:** We employed nominal group technique (NGT) to select four low-value care vignettes. Prompted by these vignettes, we conducted individual interviews with residents. We analyzed the qualitative data thematically using an inductive-deductive approach, guided by Bronfenbrenner’s social-ecological framework. This framework provided guidance to "context" in terms of sociopolitical, environmental, organizational, interpersonal, and individual levels.

**Results:** In 2022, we interviewed 19 residents from a Dutch university medical center. We identified 33 contextual factors influencing residents’ decision-making, either encouraging or discouraging low-value care. The contextual factors resided in the following levels with corresponding *categories*: (1) environmental and sociopolitical: *society, professional medical association,* and *governance*; (2) organizational: *facility characteristics, social infrastructure,* and *work infrastructure*; (3) interpersonal: *resident-patient, resident-supervising physician,* and *resident-others*; and (4) individual: *personal attributes* and *work structure*.

**Conclusion:** This paper describes 33 contextual factors influencing residents’ decision-making concerning low-value care. Residents are particularly influenced by factors related to interactions with patients and supervisors. Furthermore, organizational factors and the broader environment set margins within which residents make decisions. While acknowledging that a multi(faceted)-intervention approach targeting all contextual factors to discourage low-value care delivery may be warranted, improving communication skills in the resident-patient dynamics to recognize and explain low-value care seems a particular point of interest over which residents can exercise an influence themselves.

## Background

**Key Messages**

**Implications for policy makers**

- Policy-makers can use and target our identified factors to create a high-value care system in which individual healthcare professionals are facilitated to deliver such high-value care to their patients.
- The delivery of high-value care can be supported with policy changes influencing the broader healthcare environment and organizational factors within which individual healthcare professionals make decisions.
- Policy-makers can increase the awareness on the overusing of care culture, including the societal sentiment of patients as “more is better,’ organizational decision-making processes and standards of practice, and the supervisors’ behavior.

**Implications for the public**

Many patients receive so-called low-value care. Such care can be considered as waste; it does not benefit patients while it might do harm and stresses scarce healthcare resources. Regarding the sustainability of our healthcare system, it is important that future physicians (residents) know how to make decisions to reduce this low-value care. However, decision-making is a very complex process influenced by contextual factors. Specifically, we found that 33 contextual factors influenced residents’ decision-making concerning low-value care. As such, residents are influenced by factors related to the interaction with patients and their supervisors. Furthermore, organizational factors and the broader healthcare environment set margins within which residents make decisions. In order to provide high-value care to patients, improving communication skills in the resident-patient dynamics seems an essential point of interest over which residents can exercise an influence themselves.

Globally, the delivery of low-value care is straining the healthcare sector’s rising expenditures.^1^ Low-value care practices are defined as “care in which evidence suggests it adds no or very little benefit to patients, or the risk of harm exceeds the probable benefit, or, more broadly, the added costs of an intervention do not provide proportional added benefits.”^2^ It can thus be considered as waste that may also harm patients and stresses scarce healthcare resources. Experts estimate that ~20% of healthcare spending in Europe^3,4^ and ~30% in the United States^5-7^ is waste. Additionally, the resource cascade flowing from physicians’ decisions accounts inherently for 60%-80% of healthcare costs.^8,9^ As such, it is becoming increasingly important that physicians and, in particular, future physicians (ie, medical residents) are trained in making challenging but necessary decisions to reduce low-value care: limiting unnecessary costs while ensuring high-quality care.^10-13^

 Residency training is predominantly conducted in clinical practice, in which evidence-based medicine represents the dominant mode for today’s decision-making.^14^ Evidence-based medicine may ensure that residents act likewise and that collective healthcare spending and scarce resources are used optimally—providing evidence-based (cost)effective care and reducing care that has no added value. Grounded on an evidence-based approach, initiatives are developed targeting low-value care,^15-18^ such as the Canadian “Choosing Wisely” and National Institute for Health and Care Excellence’s “Do Not Do List.” However, thus far, these well-known initiatives have sometimes resulted in limited successes due to for example, preexisting professionals’ behavioral patterns or difficulty in having conversations with patients about avoiding a low-value service.^19-22^ Although acknowledging that evidence-based medicine is essential in clinical practice, evidence alone is apparently not always sufficient for effective decision-making regarding low-value care.^23,24^

 Decision-making is a highly complex process shaped by an interrelated set of factors.^25-29^ Bock et al^30^ have shown that several factors specifically related to residents’ social context and workplace culture influence residents’ value-based decision-making and learning. Furthermore, Lang and colleagues^31^ have pointed out that primary care physicians perceived various organizational factors, such as resources, care processes, improvement activities (eg, performance measurement), and governance, to influence the use of low-value care. Other macro-level factors that have been identified affecting low-value care decision-making include biased evidence, medical education environment, a “more is better” culture, and perverse financial incentives.^32,33^ However, currently only a limited exploration of the full scope of contextual factors influencing decision-making related to low-value care within the domains of healthcare and medical education has been performed. Investigating these contextual elements could offer a more intricate comprehension of decision-making processes in clinical practice. This can ultimately improve the educational experiences for residents during their residency training, with the aim of reducing low-value care. As such, the objective of this study is to examine the various micro- to macro-level contextual factors that residents encounter and how they experience their decision-making with regards to low-value care.

## Methods

###  Study Design and Research Instruments

 This prospective study was conducted in two steps. The first step (A) encompassed the compilation of an expert panel to advise on low-value care vignettes. We employed nominal group technique (NGT) to select clinical-practice vignettes that fulfilled the definition of low-value care, which is described as care in which evidence suggests it adds no or very little benefit for patients, or the risk of harm exceeds the probable benefit, or, more broadly, the added costs of an intervention do not provide proportional added benefits.^2^ NGT is a consensus group method in which participants share and discuss their views on a specific topic and, subsequently, rank their ideas related to this topic individually.^34,35^ We chose to develop practice-based vignettes. These vignettes were designed to serve a dual purpose: firstly, to facilitate meaningful and contextually grounded discussions during the interviews, and secondly, to frame our research population’s potential encounters with low-value care practices in a realistic and nuanced manner. The second step (B) was a qualitative semi-structured individual interview approach, prompted by the selected low-value care vignettes, to explore which contextual factors were perceived by residents influencing their decision-making. Qualitative research principles informed methodological decisions about sampling, data collection, and analysis.^36,37^ We followed the quality standards for reporting qualitative research.^38^

###  Theoretical Framework

**Figure 1:**
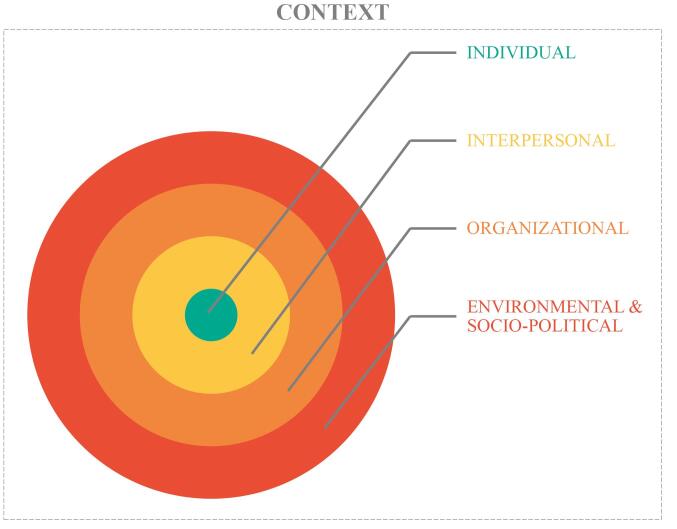
Context Approached Applying the Social-Ecological Framework.

 In this study’s data collection and analysis, we used Bronfenbrenner’s social-ecological systems framework to provide guidance to the broad concept of “context.”^39^ This social-ecological framework was initially used to explain child development, and widely used in the fields of psychology and social sciences to understand how individuals are influenced by their environment and the various systems surrounding them. The framework emphasizes the dynamic and interactive nature of human development within multiple interconnected systems, with the individual at the center. Other research used this framework to assess a variety of factors influencing decision-making.^25-27^ In our research, we applied the framework to define *context* at an individual, interpersonal, organizational, environmental, and socio-political level (see Figure 1). We conducted interviews with residents to identify which factors for every level of *context* (individual, interpersonal, organizational, environmental, and sociopolitical) play a role in residents’ decision–making concerning low-value care.

###  A. Nominal Group Technique

####  Sampling

 We recruited residents and physicians from a Dutch university medical center to participate in an expert panel. The participants were purposively sampled, using the research team’s network, for their practical knowledge and experience covering different clinical settings to reflect the clinical spectrum. We sent an email invitation for participation, including an information letter. All participants agreed with the study’s procedure and informed consent.

####  Data Collection

 In December 2021, the expert panel members received a digital three-phased questionnaire, using the Qualtrics survey software version 12.2021. The questionnaires were sent by email scattered over three weeks. In phase one and, respectively, the first questionnaire, we introduced “low-value care” as commonly described in literature^2^ and explained “contextual factors” as any factor within the social-ecological levels. Then, we posed the nominal question to the participants: “from your point of view, which low-value care is a typical example in which contextual factors influence decision-making?” Participants had to work on this question independently and privately by describing and submitting a clinical vignette.

 In phase two and, respectively, the second questionnaire, the participants’ written clinical vignettes were shared within the group. Each participant was encouraged to read and discuss the ideas one by one by providing feedback. They could also indicate if clarification was necessary. This approach ensured that all participants had equal opportunities to contribute to the panel and that consensus was reached regarding the low-value care vignettes. Since several clinical vignettes described the same type of low-value care, the research team defined four overarching low-value care themes (ie, overtreatment, ineffective care, inefficient care, and overuse of diagnostics) through extensive discussion before starting phase three.

 In phase three and, respectively, the third questionnaire, participants prioritized the generated low-value care vignettes per theme by ranking the vignettes from most to least suitable with regard to low-value care. Eventually, the ranking resulted in four vignettes, one per theme. Rerating was not needed. The final selection of the four low-value care clinical-practice vignettes represented a group consensus.

###  B. Semi-structured Interviews 

####  Sampling

 The study was conducted in the Netherlands’ Southeastern postgraduate medical education training region (in Dutch: OOR ZON). We recruited residents from different medical specialties from a university medical center and asked them to participate in individual interviews. Related to each low-value care vignette, the participants were purposively sampled using the research team’s network to cover a variation in work experience. We sent an email invitation, including a brief study description and information letter. Participants’ preferences and availability determined the interview date. Participation in the study was voluntary at all times.

####  Data Collection 

 We collected data by conducting individual, semi-structured interviews either face-to-face or online. The research team developed a semi-structured interview guide (see Supplementary file 1) based on the social-ecological framework, with specific focus on a relevant low-value care vignette as the starting point of each interview. During the interviews, the framework gave structure to the interviews as we walked through the levels of context, ie, individual, interpersonal, organizational, environmental, and sociopolitical (see Figure 1), and asked residents to identify and describe if they experienced some factors influencing their decision-making concerning the low-value care vignette. The interview guide questions related to the different levels were broad and open-ended, allowing residents to identify any factor within these levels. The factors mentioned by the residents were explored in-depth with follow-up questions during the interview. We first piloted the interview guide with a resident not participating in this study to get feedback on the order, content, and wording. The low-value care vignettes scaffolded the interviews, but residents’ experiences and explanations concerning contextual factors were not limited to them.

 The interviews were conducted between April 2022 and September 2022 by LAB (MSc, female, PhD-student), and she was assisted by FAH (BSc, female, medical student). The interviews lasted between 30 and 70 minutes. All interviews were audio-recorded and transcribed verbatim; transcripts were anonymized to ensure participants’ privacy. We offered residents the opportunity to comment on or correct transcripts concerning their interview. The lead researcher wrote detailed reflective memos regarding her insights and impressions.^40^

 We strived to reach data sufficiency regarding contextual factors in general instead of contextual factors related to a specific low-value care vignette. We reached data sufficiency after 19 interviews, meaning that no new information emerged, and sufficient data to understand and explain the influence of contextual factors on residents’ decision-making concerning low-value care had been collected.^41^

###  Data Analysis 

 We thematically analyzed the transcribed interview data using an inductive-deductive approach^37^ guided by the social-ecological framework levels. Within these levels, we identified contextual factors influencing residents’ decision-making concerning low-value care. This analysis process included different steps. First, LAB and BABE independently read and analyzed three transcripts by open coding. They labeled fragments relevant to the research focus. Subsequently, these researchers compared and discussed their coding until they reached consensus. Next, LAB coded subsequent interviews to analyze the initially developed and new codes, and we iteratively refined the initial codes. The researchers then developed a robust coding scheme by grouping codes into overarching contextual factors and discussing the coding scheme until we agreed upon a preliminary code scheme. LAB coded subsequent interviews to analyze (dis)confirmation of codes. We further refined the coding scheme during this process through frequent group discussions. Eventually, we constructed several categories by grouping contextual factors and reaching a deeper understanding of the factors influencing residents’ decision-making. All researchers reviewed the ultimate coding tree. ATLAS.ti (version 9) supported the data analysis.

###  Reflexivity 

 Our research team consisted of a PhD student trained in qualitative research (LAB); a strategic and medical educational advisor and researcher with experience in conducting research in value-based healthcare and postgraduate medical education (CYGN); a medical doctor with a particular interest in value-based healthcare (RHLH); a medical student (FAH); a medical doctor and educator experienced in qualitative research, and full professor in professional development in postgraduate medical education (WNKAvM); and a senior researcher health technology assessment (BABE). Our team included various levels of experience and fields of expertise, ranging from knowledge of clinical practice to medical education and healthcare efficiency, which was deemed necessary considering this study’s topic. The team was thoroughly involved in the interview guide development and data analysis to maximize the contributions from these different backgrounds. To minimize a possible dependency relationship (leading to socially desirable responses), the interviewers were not directly linked to the (education of) participating residents.

## Results

###  Demographics 

 The expert panel included 13 participants from both surgical (n = 5) and non-surgical specialties (n = 8). In the surgical group, 80% were male, the mean age was 49 years (range: 35-61 years), and their total clinical work experience was, on average, 20 years (range: 5-35 years). In the non-surgical group, 63% were male, the mean age was 47 years (range: 33-66), and their total clinical work experience was, on average, 17 years (range: 2-40 years). Table 1 shows the expert panel’s characteristics. The expert panel’s consensus resulted in the following four low-value care clinical-practice vignettes: (1) dual coronary angiography diagnostics; (2) ineffective frequency and loudness matching with tinnitus; (3) overtreatment of cerebral artery aneurysm; and (4) unnecessary colonoscopy. The elaborated descriptions of the vignettes can be found in Supplementary file 2.

**Table 1:** Characteristics of the Expert Panel (n = 13)

**Characteristics **	**Specialties**	
	**Surgical **	**Non-surgical **
n	5	8
Male (%)	80	63
Age, mean (years)	49	47
Range	35-61	33-66
Clinical work experience, mean (years)	20	17
Range	5-35	2-40

 Concerning the individual interviews (n = 19), 13 residents were male and six were female, the mean age was 33 years (range: 28-41 years), and the participants were, on average, in their fourth year of residency (range: 1-6 years). The duration of the residency programs of the participating residents varied from four to six years. Table 2 shows a detailed overview of the participants’ characteristics according to each vignette.

**Table 2:** Characteristics of the Interviewed Residents (n=19)

**Characteristics **	**Low-Value Care Vignette**			
	**1. **	**2. **	**3. **	**4. **
Specialty	Cardiology Cardio-thoracic surgery	Audiology	Neurosurgery Neurology	Gastroenterology-hepatology
n	7	3	5	4
Male (%)	100	67	40	50
Age, mean (years)	34	33	31	33
Range	30-36	28-41	29-32	30-37
Residency training, mean (years)	5	3^a^	3	5
Range	3-6	2-4	1-5	3-6

^a^ = One “resident” had already finished residency training for a couple of months at the time of the study. Since there were no other residents within the studied setting, we recruited this specific individual. Vignette 1 = dual coronary angiography diagnostics. Vignette 2 = ineffective frequency and loudness matching with tinnitus. Vignette 3 = overtreatment of cerebral artery aneurysm. Vignette 4 = unnecessary colonoscopy.

###  Contextual Factors

**Figure 2:**
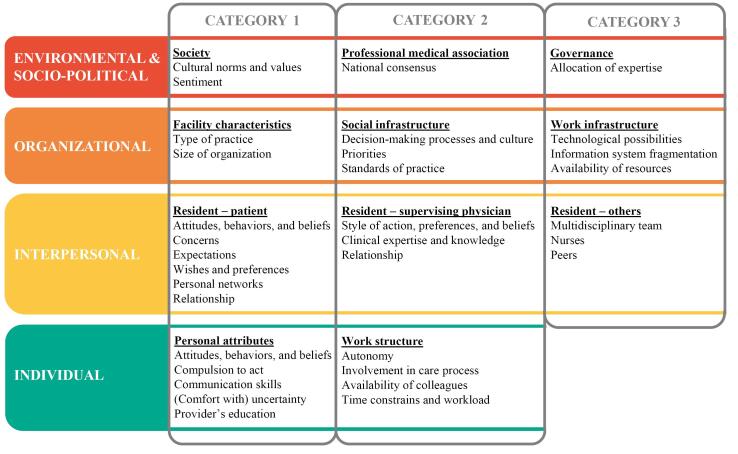
The Identified Contextual Factors With Corresponding Categories Within the Social-Ecological Levels Influencing Residents’ Decision-Making Concerning Low-Value Care. Note: The categories are underscored and in bold.

 Data analysis yielded 33 contextual factors influencing residents’ decision-making concerning low-value care. As described, we grouped these factors into categories per social-ecological levels during our analysis. The contextual factors to the various categories within the social-ecological levels are displayed in Figure 2. Overall, residents described that decision-making concerning low-value care in clinical practice could be considered situational and dynamic (ie, each case differed and could not be seen as clear-cut) in which an interplay of contextual factors interacted, albeit unconsciously, and present to a greater or lesser degree. Furthermore, residents explained that if they deemed a procedure medically justified (benefits outweighed harms)—but perhaps unnecessary—there was more space for contextual factors to influence decision-making. Residents also mentioned that depending on the perspective, ie, the individual patient or society, care was of low value or not. In the following sections, we describe the contextual factors along with the identified categories across the social-ecological levels and how they functioned as explained by the residents. We explain the categories with associated contextual factors starting from the environmental and socio-political (outer) level towards the inner levels. It should be noted that the factors of the different levels influenced each other. The contextual factors of the outer levels (organizational, and environmental and socio-political) delineated the decision-making space within which the resident could navigate. Within this space, the contextual factors associated with the interpersonal level further shaped the decision-making dynamics between the patient and resident. This interaction was subsequently affected by contextual factors related to the individual level.

###  Environmental and Socio-Political Level

 Corresponding to the environmental and socio-political level of context, the identified categories were: society, professional medical association, and governance.

####  Category 1. Society 

 Residents mentioned two society-related contextual factors influencing their decision-making concerning low-value care: “cultural norms and values,” and “sentiment.” Residents explained cultural differences in societal norms and values considering the ever-continuing care delivery. They said that Dutch cultural norms and values were somewhat more preserved in the ongoing treatment of patients compared to other countries. Furthermore, residents experienced that, depending on the cultural background, patients reportedly had a greater *“fear to be ill” *(Participant 13, indicated as P13), potentially resulting in overuse of care. Residents also noticed a sentiment in Dutch society regarding health that was sometimes challenging for their decision-making, as it shaped patients’ expectations to the extent that *“action [care delivery] is seen by default as something positive” *(P11).

####  Category 2. Professional Medical Association

 Residents mentioned one contextual factor related to the professional medical association: “national consensus.” The professional medical association played a key role in developing guidelines on the national level to guide appropriate treatment. According to residents, uniformity in care delivery across healthcare organizations would exist if there was a national consensus on the standard of practice. Although varying by discipline, residents signaled that there were limited standards of practice guidelines nationally because, for example, *“there’s no unequivocal evidence for the best practice”* (P4). As a result, *“one hospital opts for one way, another [hospital] for another”* (P10), potentially causing low-value care delivery. Residents indicated that a lack of national consensus provided space for local and/or regional organizational and individual professional preferences.

####  Category 3. Governance 

 Residents brought up one contextual factor related to governance concerning their decision-making in low-value care: “allocation of expertise.” Residents mentioned it was nationally decided* “how [hospital] care is organized”* (P3). They explained that this allocation of expertise could lead to a repetition of certain procedures. For instance, some institutions did not provide certain therapeutic interventions after initial diagnostics. This resulted in patient referral to other institutions, where again the same diagnostics were repeated prior to the intervention.

###  Organizational Level

 The identified factors were compiled into the following categories related to the organizational level of context: facility characteristics, social infrastructure, and work infrastructure.

####  Category 1. Facility Characteristics 

 Two contextual factors related to facility characteristics became apparent during data analysis: “type of practice” and “size of organization.” The type of practice significantly affected residents’ decision-making. Residents stressed that, within general hospitals, *“production is more of a thing”* (P8) and, in this regard, *“delivering procedures makes money*” (P18). As such, residents thought there was somewhat of a financial incentive for the organization, allowing overuse of care. Within university medical centers, residents explained that care delivery was characterized *“from a learning or educational point of view, a little more objectifying”* (P12). Herein, unnecessary procedures were more likely to be performed for educational purposes. Furthermore, the type of practice, but also the size of organization, determined the allocation of consultation time: *“general hospitals will always have a slightly busier outpatient clinic (…) and thus also less time for each patient. And here [university medical center] it’s a little more time, more explaining” *(P17). This time constraint was also experienced to be the case for small organizations and affected residents’ time availability to make decisions, potentially causing low-value care delivery.

####  Category 2. Social Infrastructure 

 Residents explained three contextual factors related to social infrastructure affecting their decision-making concerning low-value care: “decision-making processes and cultures,” “priorities,” and “standards of practice.” First, residents distinguished two types of decision-making processes and cultures constituted by the type of practice: a pragmatic and *“what the patient wants and thinks (s)he needs” *(P6) approach in general hospitalsversus an evidence-based and *“can we better spend our scarce time on another or more complex patient” *(P6) approach in university medical centers. Residents summarized this as *“general hospital puts the patient a little more at the center, whereas university medical center puts care as a whole a little more at the center” *(P5). Second, residents described that different organizations each had their own priorities; for example, research-focused with a potential downside: *“things [care delivery] are done but not always necessary for the patient, but it’s justified to do so”* (P19). Third, organizational standards of practice were repeatedly mentioned, which determined norms and the courses of action, such as routines, (in)formal care pathways, and protocols. According to residents, the present medical profession members formed these organizational standards of practice based on, for example, their own experiences and agreement with evidence. However, residents noticed this also contributed to low-value care because standards of practice could become habits: *“standard routine you do”* (P19) and *“we’ve always done it this way, so we’re still doing it this way”* (P7).

####  Category 3. Work Infrastructure 

 Three contextual factors related to work infrastructure were mentioned by residents influencing their decision-making concerning low-value care: “technological possibilities,” ‘information system fragmentation,” and “availability of resources.” First, residents noted that technological possibilities facilitated healthcare; however, it was perceived to also make requesting and performing diagnostics more readily accessible and more easily deployed. It also stimulated testing: *“measuring is knowing these days, and when you’ve the possibility, it’s not so neat to patients not to do it”* (P2). Second, institutional information system fragmentation was sometimes reported, resulting in duplication of non-invasive procedures. As one resident talked about a patient transferred for surgery: *“we just want to have it in the system ourselves” *(P1). Third, residents mentioned they were infrequently constrained by the availability of resources such as materials, supplies, and equipment. Though personnel capacity and available subspecialized expertise were perceived to influence decisions, but these constraints mainly led to patient delays instead of incorrect treatment.

###  Interpersonal Level

 Within the interpersonal context level, the contextual factors could be grouped into the following categories: resident-patient, resident-supervising physician, and resident-others.

####  Category 1. Resident-Patient

 Data analysis resulted in six contextual factors related to residents’ interaction with patients: “attitudes, behaviors, and beliefs,” “concerns,” “expectations,” “wishes and preferences,” “personal networks,” and “relationship.” First, each patient differed in attitudes, behaviors, and beliefs, potentially leading to a demanding, firming, or empowering patient disposition. In turn, *“you might be more inclined to undertake that additional procedure”* (P12). Patients’ attitudes, behaviors, and beliefs could be roughly characterized by their cultural background (see contextual factor: cultural norms and values) and generation: *“the younger ones [patients] ask what’s the evidence for this, why are we doing this and not that. But the older patients think the doctor knows”* (P17). Second, residents explained that patients’ concerns highly influenced their decision-making. If residents sensed that patients were concerned, they could decide to deliver care in *“therapeutic manners”* (P7). Residents stressed these situations did not feel like low-value care because they expected that patients would have consumed more care when not being reassured. Third, patients had expectations when visiting the hospital and anticipated getting diagnostic procedures or interventions: *“then try to talk them out of it off”* (P16). Fourth, patients’ wishes and preferencessometimes resulted in low-value care delivery since *“if one [patient] really wants it, then I think we’re willing to do it”* (P5). However, residents mentioned that they complied with patients’ wishes and preferences based on a why-concern rather than a what-concern. Fifth, all the above-described patients’ aspects were experienced to be framed by patients’ personal networks.The personal network could either participate in the interaction directly or indirectly (eg, patients’ family could frame their expectations in advance). Lastly, to ultimately maintain the physician-patient relationship, residents described decision-making as *“it’s a bit of give and take”* (P16); decisions favoring relationship establishment could thus lead to overuse.

####  Category 2. Resident-Supervising Physician

 Residents spoke about three contextual factors related to their interaction with supervising physicians affecting decision-making concerning low-value care: “styles of action, preferences, and beliefs,” “clinical expertise and knowledge,” and “relationship.” Residents described that supervising physicians significantly influenced their decision-making: *“in the end, I’ve to follow the policy my supervisor wants”* (P12). Residents consistently reported various supervisor’s styles of action, preferences, and beliefs: a defensive or pragmatic course of action, beliefs in novel evidence or sticking to their routines, degrees of conservatism (based on personal experience), or sensibility for patients’ wishes and preferences, for instance. Supervisor types were, in turn, shaped by their individual clinical expertise and knowledge: *“He [experienced supervisor] experienced a lot of things and many cases where things went well or didn’t go well. So he does include all [this experience] into his decisions”* (P9). Lastly, residents had a relationship with their supervisors, both formally and informally. Regarding formal relationships, to keep residents’ training on track, they said:* “one says this, the other says that. To some I will oppose, to the other I won’t” *(P4). This led to different outcomes given residents’ decision-making concerning low-value care. Regarding informal relationships, supervisors were the frames of reference in clinical practice: *“I’m educated by the people here, I learn what they do and think” *(P5). Although previously limitedly focused on low-value care, residents noticed the topic gained more attention, yet variable among their supervisors according to their educational generation.

####  Category 3. Resident-Others 

 The interaction of residents with other actors also had an influence on their decision-making concerning low-value care. These actors were: “multidisciplinary team,” “nurses,” and “peers.” Decisions could be made within a multidisciplinary team: *“you discuss together to make a trade-off in the group (...) based on their experiences and evidence” *(P10). The outcome of the decisions was thus affected by the staff composition that brought their individual identities into the interaction (see category 2 resident-supervising physician). Furthermore, experienced nurses were considered valuable in deciding the appropriate care pathway by sharing their knowledge and patient information. Lastly, residents described the interaction with peers as discussing partners with which they debated multiple matters in a low-key manner.

###  Individual Level

 Apart from the described upper levels of context, the factors related to the individual context’s level could be grouped into the following categories: personal attributes and work structure.

####  Category 1. Personal Attributes 

 Data analysis yielded five contextual factors related to personal attributes: “attitudes, behaviors, and beliefs,” “uncertainty,” “compulsion to act,” “communication skills,” and “provider’s education.” First, residents mentioned that differences in attitudes, behaviors, and beliefs, either stimulated or impeded low-value care. For example, residents’ backgrounds and personalities were explained: *“some find it harder to say no because it’s not in their personality”* (P5). We also noted varying beliefs among residents regarding the importance of healthcare costs in decision-making. Some residents mentioned that benefits versus costs did not play a considerable role when making decisions; instead, they focused on benefits versus harms.Second, we noticed residents had a compulsion to act because most residents described they wanted to satisfy and give patients the feeling that *“they’re taken seriously”* (P5) or as *“offering something for your patients, providing service” *(P18). Third, residents said their degree of communication skills contributed to explaining to patients why they did not need a specific procedure. Fourth, residents occasionally overused diagnostics to deal with their uncertainty: *“to reassure myself a bit, you don’t miss anything”* (P13). Overall, residents explained that while gaining more experience, their communication skills improved and comfort with uncertainty also increased. Finally, residents mentioned the contemporary provider’s education had an increasing tendency towards learning about healthcare value and practicing cost-consciously: *“I think we’re already much more conscious of that than the older doctors”* (P16). Thereby, some residents indicated medical education should concentrate more on improving communication skills. As such, they sometimes experienced a misalignment between their expected patient preferences with the actual patient preferences.

####  Category 2. Work Structure 

 Residents mentioned four contextual factors related to work structure that influenced their decision-making concerning low-value care: “autonomy,” “involvement in care processes,” “availability of colleagues,” and “time constraints and workload.” First, residents’ position was characterized by a dependency on superiors (eg, supervisors or multidisciplinary team professionals, see interpersonal level), which affected their decision-making autonomy. Second, there were differences in the involvement in care processes related to residents’ specialties. Surgical-specialty residents mentioned they were less involved in patients’ long-term follow-up, which limited long-term outcome feedback on their actions. Third, residents brought up that the availability of colleagues affected their decision-making concerning low-value care. During night shifts, for example, the possibility of consulting more knowledgeable colleagues was limited, and residents needed to make decisions independently, linked to contextual factor: uncertainty. Fourth, residents reported they occasionally experienced time constraints and workload during consultation time. High workload resulted in limited time available for an explanation, which sometimes led to giving in to the patient*: “if there’s time pressure and I’m already behind [in schedule], then it may be the case I’ll say: okay, we do it [requesting diagnostics]”* (P16).

## Discussion

 This study explored which contextual factors influenced residents’ decision-making concerning low-value care. We unraveled 33 contextual factors influencing residents’ decision-making, either encouraging or discouraging low-value care. The identified factors ranged from the level of the individual resident (eg, compulsion to act and communication skills), to interpersonal (eg, patients’ expectations and supervisors’ style of action, preferences, and beliefs), to organizational (eg, standards of practice and organizational decision-making processes and culture), and to the environmental and socio-political system (eg, societal sentiment and allocation of expertise). We found that residents experienced decision-making as highly situational, in which an interplay of contextual factors occurred. Roughly said, the interplay consisted of the resident—connected to their supervisor—and the patient bringing their personal and/or professional identities into the medical encounter’s interaction. This interaction, in turn, is affected by factors surrounding the resident and the work environment. Organizational factors and the broader environment and the socio-political system set margins in which residents make decisions concerning low-value care.

 A prior study has identified key factors affecting low-value care on a macro-level (ie, environmental and socio-political level) by interviewing organizational leaders, policymakers, low-value care researchers, and project leaders.^32^ This study revealed the following factors: payment system, pharmaceutical and medical device industry, fear of malpractice litigation, biased evidence and knowledge, medical education, and a “more is better” culture. However, we discovered fewer factors on the national level, probably due to the selection of professionals interviewed. The residents in our study were possibly less aware of issues related to the payment system or the industry. Furthermore, a literature study identified contextual factors influencing meso-level (ie, organizational level) decision-making in “value” (cost and quality), such as organizational characteristics, interest groups within the organization, governance and leadership, and government and regulatory factors.^29^ Another literature study focused on identifying drivers of overuse and underuse of medical care in systems worldwide on *all *levels.^42^ The authors concluded that the provision of care is influenced by the resources available, social and political contract, state of scientific knowledge, configuration and capacity of the system, and financing mechanisms. Regarding both literature studies, several contextual factors influencing organizational decision-making are also reflected in our results; however, our analysis yielded contextual factors more directly related to the individual decision-maker, ie, the resident. These differences can be explained by the differences in study design, and in particular, our participants’ position and perspectives.

 Overall, we identified contextual factors over a wide range of “context” influencing residents’ decision-making concerning low-value care. In light of reducing low-value care delivery, our results illustrate at each level of context the importance of the “culture” within which decisions are made. This encompasses the societal sentiment and culture but, particularly, the organizational and residents’ supervisors (including multidisciplinary team) culture. This finding was also reported in previous studies.^29,30,42,43^ For example, Gupta and Moriates^43^ stated that the culture within healthcare microsystems needs to be meaningfully addressed to change clinicians’ behaviors to deliver high-value care. Moreover, this addresses the importance of the hidden curriculum by which residents learn.^44,45^ In fact, the hidden curriculum transfers norms, values, and practices of the workplace environment to residents through its culture and interactions in which supervisors are primarily the reference frame. Although the topic of low-value care (and closely related concepts) is gaining awareness and attention, it should be noted that senior physicians might have received little to no specific training in this topic. In such an environment, residents may find it challenging or impossible to incorporate high-value care principles into practice unless this culture is acknowledged and addressed.

 Interestingly, we found that the interviewed residents generally emphasized contextual factors related to the interpersonal context’s level. The interpersonal level addresses the influence of social networks in decision-making—roughly explained as professionals and the healthcare delivery system on one side and patients (and their families) on the other. Recently, we conducted a social network analysis concerning residents’ value-based decisions, balancing health outcomes against healthcare costs.^30^ Results indicated that residents experienced patients as highly influential in their decision-making. Patients and their families could pressure the resident with a demanding and compelling attitude, leading to unnecessary procedures. In addition, a qualitative evidence synthesis reported that patient expectations are a significant factor limiting the reduction of low-value care.^33^ These findings align with our results that patients were perceived as highly influential. Accordingly, patients’ expectations, wishes, and preferences can drive low-value care.^22,33,46,47^ However, it is important to bear in mind that the drive to act and do something for the patient, regardless of a clear medical need, might not always lead to actual patient satisfaction. As such, research indicated that patient satisfaction is linked to communication, follow-up, clear diagnosis, shared decision-making, and acknowledgment that their symptoms are real.^48,49^ Thus, regarding avoiding low-value care, it seems important to improve residents’ communication skills—asking patients about their expectations, wishes, and preferences, and creating a clear follow-up plan or discussing a conservative approach with the patient. Indeed, previous research has shown that a lack of communication skills was found to be a significant barrier to low-value care reduction.^33^

 While improving communication skills in general, it is also important to distinguish between low-value care practices based on evidence quality and recommendation “strength” specifically.^50^ For practices with high-quality evidence against them, residents should actively discourage these options rather than present it as a valid option during treatment discussions with patients. This involves understanding and validating the patient’s reasons for requesting such care, while explaining why certain practices are not recommended. More commonly however, in cases with more conditional recommendations, residents should engage in shared decision-making. If a patient mentions a low-value practice, the resident should communicate the existing evidence regarding this practice and co-create a suitable solution in an informative, constructive dialogue with the patient.

## Implications for Practice

 It is important to note that residents recognized that it could differ whether care was of low value for the individual patient or society. Moreover, Verkerket al^51^ defined low-value care as follows: “care that is unlikely to benefit the patient given the harms, cost, available alternatives, or preferences of the patient.” In this, care is of low-value when meeting both the patients’ and societal perspectives. Nevertheless, the residents included in this study described that they occasionally experienced tension between these two perspectives in clinical practice. In their view, the value of care could differ according to the individual patient’s or society’s perspective, eg, “unnecessary” colonoscopy. Based on the history taking, the patient’s symptoms align with irritable bowel syndrome, and consequently, according to the available guideline, there is no indication for performing a colonoscopy. Nevertheless, a demanding patient who is very worried insists on a colonoscopy (high value from patient’s perspective). In this example, the resident might feel pressured into ordering a colonoscopy, which is also from a societal perspective *unnecessary* and thus of low value.

 Furthermore, although we identified a broad range of contextual factors, it is evident that an individual resident has limited influence on several factors related to the organizational-, environmental-, and socio-political level. When seeking to understand and improve decision-making concerning low-value care, we will, therefore, address an essential factor over which residents can exercise influence themselves. Since the patient-clinician dynamic is ultimately where low-value care manifests,^51^ it seems highly beneficial for medical education to focus on communication. Residents’ communication skills seem critical in multiple ways. Targeting patient expectations through improving residents’ communication skills may provide an opening for the patient to explicate and discuss their preferences and wishes. Sypes and colleagues^52^ reported that engaging patients within the patient-clinician dynamic led to a reduction of low-value care. Besides, not all patients’ problems and concerns require a medical action from the resident but rather demand communication skills to explain to the patient why a test or treatment is unnecessary and may even be harmful. Literature confirms that improving clinicians’ communication skills is a highly pivotal component for the reduction of low-value care.^53^

###  Recommendations for Future Research

 We examined contextual factors affecting residents’ decision-making concerning low-value care. Future research could focus on exploring the effect of contextual factors related to specific clinical settings and decision-making processes, such as diagnosis, intervention, evaluation, and counseling. In addition, investigating the degree to which the factors influence decision-making is warranted; some factors may be of greater influence than others. Therefore, we encourage researchers to quantify the importance of the factors related to specific settings and decision-making processes. Furthermore, conducting a comprehensive complementary study into the interconnectedness among these factors will enhance our understanding of the contextual elements that shape residents’ decision-making in the context of low-value care. Finally, future research could explore the perspectives of attending or faculty physicians.

###  Strengths and Limitations 

 We identified contextual factors influencing the individual decision-maker over the wide range of “context”: individual, interpersonal, organizational, environmental, and sociopolitical levels. Our identification and description of contextual factors is a meaningful addition to the body of literature describing determinants and drivers of low-value care and contextual factors influencing decision-making.^29,32,33,42,54-56^ A strength of this present study is the richness of data ranging from factors related to the individual decision-maker to the socio-political system from the perspective of residents working in clinical practice. We used the social-ecological framework to provide guidance in our study. Although physicians’ role is central to a wide range of decision-making theories,^14,57^ no single theory did explicate decision-making in the clinical practice as a whole, fitting the broadness of context.^58^ Nevertheless, previous studies have already applied the social-ecological framework (initially used to explain child development) to assess various factors influencing decision-making.^25-27^

 This study also has its limitations. First, we conducted the expert group online due to the COVID-19 pandemic, while NGT participants usually meet face-to-face. Considering participation on the expert panel during the pandemic, we chose a three-phased self-completed questionnaire to provide flexibility for participants. This approach potentially led to less interaction, although we extensively encouraged participants to discuss each other’s vignettes. Moreover, we acknowledge that the selected low-value care vignettes may not be considered as low-value from all perspectives (see the explained perspective issue on classifying care as low-value). Besides, the prevalence of the low-value care vignettes was unknown. Nevertheless, the vignettes’ purpose was to scaffold the interviews and get the conversation started on this study’s broad topic; residents were not limited to talking solely about the vignettes. Another limitation of this study is that the multifaceted nature of low-value care discussions revealed a complex interplay of influencing factors beyond the scope of residents themselves. This may have made it challenging for residents to precisely assess the specific impact of their knowledge and competence regarding low-value care. Lastly, the study’s results were influenced by the study setting and participants’ perspective, which limits the findings’ transferability and generalizability. Although the included participants are physicians in a Dutch university medical center, they also have experience in working in a general hospital. In addition, several results are specific to the Dutch clinical practice, but most of the identified contextual factors influencing decision-making may be applied to other clinical practices of high-income countries when sharing similar healthcare system characteristics. However, the mechanisms of these contextual factors on low-value care could differ.

## Conclusion

 In this study, we identified 33 contextual factors influencing residents’ decision-making concerning low-value care, residing in context levels from the individual decision-maker to the socio-political system. This decision-making was experienced as an interplay of many contextual factors, resulting in encouragement or discouragement of low-value care delivery. Residents are particularly influenced by factors related to interactions with patients and supervisors. Furthermore, organizational factors and the broader environment set margins within which residents make decisions. While acknowledging that a multi(faceted)-intervention approach targeting all contextual factors to discourage low-value care delivery may be warranted, improving communication skills in the resident-patient dynamics to recognize and explain low-value care seems a particular point of interest over which residents can exercise an influence themselves.

## Acknowledgements

 We would like to thank the expert panel members and the residents for their time and explanations.

## Ethical issues

 We informed all participants of the study’s purpose, participation mode, and confidentiality. All participants provided consent for participation. Participation was entirely voluntary, and data was handled confidentially and anonymously. The Medical Ethics Review Committee of Maastricht University Medical Center+ and Maastricht University (ref. no. 2021-2819) granted the study’s ethical approval on July 19, 2021.

## Competing interests

 Authors declare that they have no competing interests.

## Funding

 The authors have not declared a specific grant for this research from any funding agency in the public, commercial or not-for-profit sectors.

## Supplementary files

Supplementary file 1. Interview Guide.

Supplementary file 2. Final Selection of the Low-Value Care Clinical-Practice Vignettes.
