# Supplementary material for: Unravelling Low-Value Care Decision-Making: Residents’ Perspectives on the Influence of Contextual Factors
Source: Int J Health Policy Manag. 2024 Apr 6;13:7907. doi: 10.34172/ijhpm.2024.7907 (PMC11608293; doi:10.34172/ijhpm.2024.7907)
Supplement: Supplementary file 1 — Interview Guide. [file ijhpm-13-7907-s001.pdf]

Article title: Unravelling Low-Value Care Decision-Making: Residents' Perspectives on the Influence of Contextual Factors

Journal name: International Journal of Health Policy and Management (IJHPM)

Authors' information: Lotte A. Bock[1,2]*, Cindy Y.G. Noben[1], Roel H.L. Haeren[3], Florine A. Hiemstra[4], Walther N.K.A. van Mook[1,2,5], Brigitte A.B. Essers[6]

[1]Academy of Postgraduate Medical Education, Maastricht University Medical Centre, Maastricht, The Netherlands. [2]School of Health Professions Education, Maastricht University, Maastricht, The Netherlands.

[3]Department of Neurosurgery, Maastricht University Medical Centre, Maastricht, The Netherlands.

[4]Faculty of Health, Medicine, and Life Sciences, Maastricht University, Maastricht, The Netherlands.

[5]Department of Intensive Care Medicine, Maastricht University Medical Centre, Maastricht, The Netherlands.

[6]Department of Clinical Epidemiology and Medical Technology Assessment, Maastricht University Medical Centre, Maastricht, The Netherlands.

***Correspondence to:** Lotte A. Bock; Email: lotte.bock@mumc.nl

Citation: Bock LA, Noben CYG, Haeren RHL, Hiemstra FA, van Mook WNKA, Essers BAB. Unravelling low-value care decision-making: residents' perspectives on the influence of contextual factors. Int J Health Policy Manag. 2024;13:7907. doi:10.34172/ijhpm.2024.7907

Supplementary file 1. Interview Guide

Note: The specific low-value care clinical-practice vignette and interview's purpose was sent to the interviewee, prior to the interview. Furthermore, oral consent was given before the start of the interview.

Introduction

- Thank you for agreeing to speak with me about the influence of contextual factors on decision-making regarding low-value care.
- Your input as a clinician is very useful to gain further insight into this topic. I'm a PhD student and I have a background in healthcare policy and management, so I'm not a clinician. I'm very interested in your views, experiences, and insights. There are no right or wrong answers. That's also why we'd like you to share information you feel is relevant.
- Brief explanation of the interview (participants are informed in advance of the entire research's purpose).
    - We would like to get an understanding of the influence of contextual factors when making decisions regarding low-value care.
    - Explanation of the social-ecological framework's individual, interpersonal, organizational, environmental, and sociopolitical levels
    - The low-value care vignette is used for guidance within the interview but feel free to give your opinion and experiences beyond the vignette.
- May be overlap, some answers repeated.

- Any questions before we start?

Discussing influence of contextual factors

- To start, I'd like to get an idea of your process in decision-making for *specific low-value care clinical-practice vignette*. Could you walk me through the steps involved in the decision-making process, which information do you use? Besides clinical information, which contextual factors are involved within your decision-making regarding *specific low-value clinical-practice vignette*.
    - Deeply explore mentioned factors
- Continuing the interview by using the framework's levels:
    - Individual
    - Interpersonal
    - Organizational
    - Environmental
    - Sociopolitical
- Thank you for answering the questions.

Ending

- Is there anything else you'd like to say or expand on?
- Who else would be interesting to talk to?
- You will receive the transcribed transcript if desired.
- Thank you very much for your time.
