# Supplementary material for: Unravelling Low-Value Care Decision-Making: Residents’ Perspectives on the Influence of Contextual Factors
Source: Int J Health Policy Manag. 2024 Apr 6;13:7907. doi: 10.34172/ijhpm.2024.7907 (PMC11608293; doi:10.34172/ijhpm.2024.7907)
Supplement: Supplementary file 2 — Final Selection of the Low-Value Care Clinical-Practice Vignettes. [file ijhpm-13-7907-s002.pdf]

Article title: Unravelling Low-Value Care Decision-Making: Residents' Perspectives on the Influence of Contextual Factors

Journal name: International Journal of Health Policy and Management (IJHPM)

Authors' information: Lotte A. Bock[1,2]*, Cindy Y.G. Noben[1], Roel H.L. Haeren[3], Florine A. Hiemstra[4], Walther N.K.A. van Mook[1,2,5], Brigitte A.B. Essers[6]

[1]Academy of Postgraduate Medical Education, Maastricht University Medical Centre, Maastricht, The Netherlands.

[2]School of Health Professions Education, Maastricht University, Maastricht, The Netherlands.

[3]Department of Neurosurgery, Maastricht University Medical Centre, Maastricht, The Netherlands.

[4]Faculty of Health, Medicine, and Life Sciences, Maastricht University, Maastricht, The Netherlands.

[5]Department of Intensive Care Medicine, Maastricht University Medical Centre, Maastricht, The Netherlands.

[6]Department of Clinical Epidemiology and Medical Technology Assessment, Maastricht University Medical Centre, Maastricht, The Netherlands.

***Correspondence to:** Lotte A. Bock; Email: lotte.bock@mumc.nl

Citation: Bock LA, Noben CYG, Haeren RHL, Hiemstra FA, van Mook WNKA, Essers BAB. Unravelling low-value care decision-making: residents' perspectives on the influence of contextual factors. Int J Health Policy Manag. 2024;13:7907. doi:10.34172/ijhpm.2024.7907

Supplementary file 2. Final Selection of the Low-Value Care Clinical-Practice Vignettes

Vignette 1.<br>A diagnostic coronary angiogram is performed in a healthcare organization where no (direct) intervention can occur. If, however, done, patients are discussed within the cardiac team and nowadays often receive another diagnostic coronary angiogram at another location afterward, accompanied with possible interventions or measurements. Subsequently, they are discussed in a cardiac team for the second time.
Vignette 2.<br>In the case of patients with tinnitus complaints, a 'frequency and loudness matching' is commonly used by otolaryngologists and audiologists to determine the nature and loudness of the tinnitus as carefully as possible. However, tinnitus' nature and character have no proven relationship with the severity of symptoms nor any influence on the treatment options. Despite the limited clinical relevance, the examination is often performed.
Vignette 3.<br>A Dutch patient has made a Prescan (total body scan) in, for example, Germany, on which a 2mm middle cerebral artery aneurysm was discovered. Thereafter, the patient is counseled in a Dutch hospital, where it is explained that the risk of treatment is greater than the risk of aneurysm bleeding or another event. The patient indicates that h/she cannot deal with the idea of having a potential 'ticking time bomb' and desires treatment. The aneurysm is treated by endovascular coiling or neurosurgical clipping based on the psychological indication.
Vignette 4.<br>A 48-year old patient has had abdominal symptoms of pain and discomfort in episodes for many years, accompanied by an altered defecation pattern of diarrhea alternating with constipation. During periods without abdominal symptoms, the defecation pattern is entirely normal. Furthermore, there are no alarm symptoms such as blood loss or weight loss. Based on the anamnesis, the patients' symptoms align with a functional disorder, namely Irritable Bowel Syndrome (IBS). The patient is worried and requests to perform a colonoscopy. Based on the IBS-guideline, there is no indication for a colonoscopy: no alarm symptoms, age < 55 years, and complaints explained entirely by IBS. However, the clinician still decides to accept the patient's request to perform a colonoscopy.
